# Stress, Depression and Coping among Latino Migrant and Seasonal Farmworkers

**DOI:** 10.3390/ijerph10051815

**Published:** 2013-05-03

**Authors:** Sloane Burke Winkelman, Elizabeth H. Chaney, Jeffrey W. Bethel

**Affiliations:** 1 Department of Health Sciences, California State University, Northridge, CA 91330, USA; 2 Department of Health Education & Behavior, University of Florida FLG 12, Gainesville, FL 32611, USA; E-Mail: bchaney@ufl.edu; 3 College of Public Health and Human Sciences, Oregon State University, Corvallis, OR 97331, USA; E-Mail: Jeff.Bethel@oregonstate.edu

**Keywords:** Latino, migrant and seasonal farmworkers, stress, depression, coping

## Abstract

Research shows that one in four migrant farmworkers experienced an episode of one or more mental health disorders such as stress, depression, or anxiety in their lifetime. The purpose of this mixed methods study was to explore experiences and perceptions related to stress and depression among Latino migrant and seasonal farmworkers (MSFWs), and to identify their coping behaviors for dealing with these mental health conditions. Using a mixed methods research approach, three focus group interviews of a sample of Latino MSFWs (N = 29) were conducted and a quantitative survey was implemented (N = 57) at community sites in eastern North Carolina. Four major themes emerged from the focus group data: (1) physical stress related to working conditions; (2) mental stress related to family situations, work environment, documentation status, and lack of resources; (3) depression related to separation from family and the lack of resources; and (4) use of positive and negative mechanisms for coping with stress and depression. A discussion of these themes, results from the survey findings, implications for intervention and outreach programs, along with recommendations for further research, are provided.

## 1. Introduction

An estimated 3–12 million migrant and seasonal farmworkers (MSFWs) currently live in the U.S., the majority of whom are natives of Spanish speaking countries, mostly from Mexico [1,2,3,4,5,6]. Nearly 60% of MSFWs live below the poverty level, with half earning less than $7,500 annually [1,2,3,4]. Most farm work is seasonal and sporadic, limiting workers’ ability to earn wages when the weather is bad, when crops are not ripe or ready for harvest, when they are sick, or while they are traveling to their next job [1,2]. They are underinsured or uninsured employees in an extremely hazardous vocation, providing essential services in support of a multi-billion dollar agricultural industry in the U.S. [4]. Many of these farmworkers are forced to leave their families behind in search of work, and experience the stress associated with separation from family and friends, language barriers, discrimination, and exploitation [6,7,8,9,10,11,12,13,14,15]. MSFWs have significantly poorer health than other worker populations, with many of them suffering from occupational injuries, chronic pain, heart disease, cancer, and conditions related to pesticide exposure. In addition, many suffer from mental health issues [14,16,17,18]. 

Recent studies have documented the high prevalence of stress, anxiety, depression, or substance abuse among the millions of MSFW in the U.S., with as many as 1 in 5 having an episode of one or more of these mental health disorders in their lifetime when including substance abuse [10,11,12,13,14,15,19]. Other research indicates that 25–55% of MSFW report having had clinically significant levels of depressive symptoms in the past week [10,12,20,21,22,23,24] and 17–39% of MSFW experience significant anxiety that may impair functioning [15,20,24]. Common stressors identified in previous research include documentation, immigration, discrimination, marginalization, being away from friends and family, rigid word demands, poor housing, and poor pay [12,21]. 

Some research has shown that stressed or depressed individuals in the general population are more likely to pursue negative behaviors such as drug or alcohol use, tobacco use, and high-fat diets as coping mechanisms, compounding the potential for undesirable consequences, such as domestic violence [16,17,18,25,26,27,28,29,30,31]. Conversely, other studies in the general population have shown that persons who deal with their stress and depression through positive coping behaviors, such as pursuing social support, relaxation techniques, and exercise, have better outcomes related to their health and quality of life [32,33].

The relationship between ethnicity, culture, and mental health issues is well documented [34,35,36,37,38,39,40]. While studies of stress and depression provide interesting and useful information regarding this issue for the general population, at time of this publication, no research was found which specifically examined the interaction of stress and depression and coping behaviors among MSFW. The purpose of this research study was to explore stress and depression among Latino MSFW and discover what, if any, coping mechanisms they are pursuing to ameliorate these serious mental health issues. 

## 2. Methods

### 2.1. Qualitative Participants and Methodology

Participants were recruited during the fall of 2008 from a three-county agricultural area in eastern North Carolina that has some of the highest MSFW populations in the state [3]. Potential participants were informed of the study through a trusted community gatekeeper known to the researchers via a migrant community clinic the researchers are affiliated with. The gatekeeper, though not an author, was listed as a research assistant on the University’s IRB application. The gatekeeper did not complete IRB training or certification. To assist with the recruiting, the gatekeeper was provided with Spanish versions of a brief description of the study and a participant informed consent form; however, due to literacy issues and the sensitive subject matter, these written materials were not distributed to the target population. Recruitment was conducted by the gatekeeper through word-of-mouth at migrant camps, trailer parks, churches, community mercados, and soccer teams. Participants were required to be adults (18 or older) who were Latino MSFW currently working in one of the three counties. The focus group interviews were conducted at either the participants’ place of employment or at a location in the community that was convenient to those participating (a family owned restaurant after closing hours and a community migrant serving clinic room when the clinic was closed). The final sample included 29 participants. Fifteen of the participants were females and 14 were males, and their ages ranged from 18 to 83, with an average of 35 years of age. On average, they worked 8.8 h per day, 5.6 days per week, 9 months per year. Ten were single, 18 were married, and one person was widowed. All of the participants reported that their spouses worked, although some of them were employed outside of the U.S. Housing included living in trailer homes or on shared Migrant Farmworker temporary housing “camps”. The number of children they had ranged from none to 14, with an average 2.4. These participants indicated that while they worked in agricultural farming (tobacco and sweet potato crops), their children were cared for by their spouse, family in Mexico, sister, aunt, or day care services. Some reported that their children (even young ones) were not living with them, and were in their home country of Mexico or Guatemala.

Three focus group interview sessions (focus group 1, n = 7; focus group 2, n = 10; focus group 3, n = 12) were conducted by the research team over a four-week period in the three eastern North Carolina counties during the fall of 2008. The moderator conducted all focus group sessions in Spanish. At the beginning of the sessions, verbal informed consent was obtained from participants. Verbal consent, as approved by the university’s Institutional Review Board, was used to ensure the confidentiality of undocumented residents. 

The focus group interviews followed a semi-structured format, using eight open-ended questions developed by the researchers, to guide and encourage discussion regarding stress, depression, and coping mechanisms. With each focus group, the moderator probed participants’ responses, if necessary, to further explore the question topics. 

### 2.2. Quantitative Participants, Methodology and Instrumentation

A small convenience sample of MSFWs (N = 57) was recruited from an eastern North Carolina migrant farmworker camp to participate in the study during the spring, 2009. The quantitative participants were not the same participants as the qualitative participants. Each participant was given a $10 gift card as incentive for participating. Three bilingual, community gatekeepers (distinct from our qualitative research gatekeeper) administered the instrument to participants at a large meeting room located in a migrant farmworker camp. The gatekeepers were trusted individuals in this population, and were trained in survey administration. An informed consent statement was read in Spanish to the pilot study group, prior to administration of the instrument, and all of the participants agreed to the statement and participated. For literacy and comprehension considerations, the survey was read aloud to all participants in a group format. Some participants could complete the survey themselves, others required assistance from one of the bilingual researchers. Responses were kept confidential, and participants were asked not to put any identifiable information on the surveys. Table 1 provides a complete list of survey respondent characteristics. 

**Table 1:** Demographic and occupational characteristics of survey respondents.

Characteristic	Percent (n)
Gender	
Male	91.2% (52)
Female	8.8% (5)
Age (years)	
20–30	31.4% (16)
31–40	27.5% (12)
>40	41.2% (21)
Education (years)	
<12	58.8% (30)
≥12	41.2% (21)
Race/Ethnicity	
Hispanic/Latino	96.3% (52)
Other	3.7% (2)
Time in U.S. (years)	
≤5	61.4% (35)
>5	38.6% (22)
Total number in household	
≤5	51.1% (23)
>5	48.9% (22)
Income in past year (monthly)	
<$1,000	68.8% (33)
≥$1,000	31.3% (15)
Part of previous year worked	
All or most	43.6% (24)
≤half	56.4% (31)
Hours worked (daily) in past year	
<8	24.6% (14)
≥8	75.4% (43)

A culturally sensitive survey instrument was created by the researchers to assess stress, depression, and coping behaviors in Latino MSFWs [41]. Informed by the results of the focus group, researchers utilized a comprehensive instrument design framework designed by Dillman to develop and pilot test the instrument [41]. A principal component analysis with direct oblimin rotation determined item measurement. Alpha reliability coefficients were calculated for subscales. Five factors related to stress, depression, and coping were extracted: Financial, Family Stress; Physical, Work, Societal Stress; Lack of Documentation and Resources; Depression; Locus of Control. This resulted in a final 90-item survey instrument, with seventy-five items measuring stress, depression and coping, and fifteen demographic questions. 

### 2.3. Data Analysis: Qualitative

After each focus group interview session, the translator translated verbatim the audiotaped comments of the participants into an English transcription. To ensure accuracy and completeness, these transcriptions were then reviewed, along with the transcription notes, by a member of the research team who is fluent in Spanish, and any necessary small additions of words omitted or edits were performed to ensure the transcriptions were verbatim to the audio recordings. 

Once the transcriptions were in their final form, their content was analyzed by one member of the research team experienced in qualitative research methodology for emergence of themes. The qualitative data analysis process involved four main steps: immersion, coding of data, creation of categories, and identification of themes. 

First, the transcriptions were reviewed multiple times to allow the researcher to become immersed in the data. Second, the researcher labeled the transcribed interview data using QSR’s NVivo, a qualitative data analysis software program, with codes based on thematic representation of the meaning of the participant’s comment. Third, using The Comparative Analysis Method [42], the codes were categorized into major topic areas based on their likeness. 

Lastly, the researcher completed the data analysis process by identifying themes that emerged from the data by continuing to apply the Comparative Analysis Method [42,43]. The research team then met as a group to discuss the thematic findings and discuss any discrepancies. 

Researchers consulted the COREQ (Consolidated Criteria for Reporting Qualitative Data Research) a 32 item checklist for reporting of this data [44]. In response to this criteria, none of the authors facilitated the focus groups or survey implementation. All focus groups and surveys were facilitated by respective gatekeepers. The qualitative gatekeeper is a community health worker who does not hold any advanced degrees. The gatekeepers are experienced in conducting focus groups and are trusted members of the community. A member of the research team (the first author) spent one hour training all gatekeepers on the research protocol. The researcher met all four gatekeepers for the first time for this research study. Gatekeepers were referred by a community contact of the first author. The gatekeepers did not disclose any biases or prior familiarity with the research content. Gatekeepers were not listed as research assistants on the University’s IRB application, however—all gatekeepers were trained on the research protocol and implementation.

### 2.4. Data Analysis: Quantitative

For the quantitative data, univariate analysis (*i.e.*, percents) was performed to describe the demographic and occupational characteristics of the study population as well as the frequency of behaviors to cope with stress. Bivariate analysis (*i.e.*, chi-square test and Fisher exact test) was performed to examine the associations between the top five stress coping behaviors identified in univariate analysis and demographic and occupational characteristics, respectively. Analyses were conducted using Stata version 12 (Stata Corp LP, College Station, TX, USA).

## 3. Results and Discussion

### 3.1. Qualitative Results

Four themes emerged from the focus group interview data: (1) physical stress related to working conditions; (2) mental stress related to family situations, work environment, documentation status, and the lack of resources; (3) depression related to separation from family and the lack of resources; and (4) use of positive and negative mechanisms for coping with stress and depression.

#### 3.1.1. Physical Stress Related to Working Conditions

Participants stated they felt physical stress at work. They expressed that their hands, feet, and eyes were tired. Sometimes they felt tired from standing all day at work. Some shared that their back, neck, arms, or head hurt and that they had tension in their head and neck. One participant commented that even when he sits down after work, his feet still hurt. Some participants just spoke of being tired in general. Others reported that they were not given breaks to alleviate being tired, and that they worked too many hours per day. An issue that came up was the lack of portable toilets on job sites, especially for women. Others felt physically stressed by the extreme temperatures during the seasons, such as heat from the sun in the summer and coldness in the winter. One participant commented that the cold makes his joints sore and it is therefore harder to work.

“My hands are tired. Working fast makes my hands tired. [Laughing] I dream of sweet potatoes on a conveyer belt.”
“My back hurts a lot, but, I haven’t had a medical check-up in a long time. Eight hours of [lifting heavy materials] hurts me.”
“They [the bosses] are always on our backs. The bosses are breathing down our necks at work. A solution would be … to stick to their promise of giving us more breaks.”

#### 3.1.2. Mental Stress Related to Family Situations, Work Environment, Documentation Status, and the Lack of Resources

Mental stress was another theme that emerged, with sub-themes for mental stress related to the participants’ family situations, work environment, documentation status, and lack of resources.

*Related to Family Situations.* Participants expressed that various issues caused them mental stress, but the recurring sub-themes centered on family situations. Specifically, they reported experiencing stress related to not being able to see family and children that were back in their home countries, or stress from family responsibilities, including taking care of their spouses and children, cooking, and having to engage in housework or childcare after a long day of working and commuting. 

“My wife is here but my mother is in Guatemala. With no papers, it is too hard to get back and forth. I am worried about my children as they are back in Guatemala. I have been apart from them for five years now.” 

*Related to Work Environment.* Stress related to the negative environment in which they worked was also found to be a sub-theme for mental stress among the study sample. Participants commented about the stress caused from their bosses being too demanding, such as having unreasonably high production standards or expecting everything to be perfect. Many of them also said they did not feel comfortable confronting their bosses about these issues. One participant also noted that having his employer communicate to him in English (instead of Spanish) was stressful. 

*Related to Documentation Status.* Another mental stress sub-theme that was found was related to lack of documentation to reside or work legally in the United States. Participants indicated that this caused them to worry about law enforcement and being deported back to Mexico or Guatemala. In addition, some respondents, particularly women, expressed concern over not having a driver’s license, and consequently not being able to drive. 

*Related to the Lack of Resources*. Lastly, as a final sub-theme of mental stress, some of the participants spoke about the stress related to their lack of resources, including not having a consistent or permanent job, savings, or extra money. Several of them also worried about not being able to send money back home to family in Mexico or Guatemala. A few participants commented that they did not know of resources in the community for mental health issues. One participant commented that a friend’s son (a Latino migrant farmworker) had just committed suicide a few months prior to the focus group. Participants know of the migrant clinic in the area, but mental health services is not provided due to limited resources.

“There is stress when the job is almost finished and there may not be more work.” 

#### 3.1.3. Depression Related to Separation from Family and the Lack of Resources

Among the study participants, more women than men responded to the focus group questions related to depression. Most respondents indicated that they were frequently depressed, often to the point of crying. Some stated that they felt persistent sadness because they were separated from their families back in their home countries, whom they missed badly. Others expressed feeling hopeless because they do not have enough money to pay their bills. For a few of the participants, their depression caused them to have sleeping problems.

“[My co-worker] plays too much music and the music causes memories. The memories are stressful as they remind me of my children back in Guatemala and that makes me sad.”
“I feel sleepy, then feel bad that I am not doing anything. I feel depressed. I can’t drive.”
“I don’t have enough money to pay the bills. Makes me feel hopeless and want to cry.”

#### 3.1.4. Use of Positive and Negative Mechanisms for Coping with Stress and Depression

Participants reported practicing both positive and negative coping behaviors. Positive coping behaviors identified by the participants included spending time with their friends, playing with their children, dancing, listening to music, getting extra sleep, taking a hot shower, drinking cinnamon tea, joking, setting goals, talking to a counselor, and consulting with a physician. In terms of negative behaviors, some reported that they coped by drinking beer, eating, playing the lottery, or refraining from talking about it or acknowledging it. Neutral behaviors (designated neutral by the researchers) were also mentioned as ways they attempt to ameliorate stress and depression, such as watching television (soccer and novellas) and seeking assistance in getting a permanent driver’s license. 

To feel less physically stressed, participants commented that they would sit, place their feet up, or take a hot shower, once they came home from work. A few people also commented that they enjoyed sleeping to recover from physical stress, and that getting enough sleep helped to relieve physical tension.

“Sleeping 8 h a day before working helps.”
“I set a goal and try to accomplish it. I first cry, then set a goal to get out of the depression.”
“Two or three beers relax me!”
“I relax by watching TV. But if my soccer team loses, that makes me even more stressed.”
“I just hold it in.”

### 3.2. Quantitative Results

#### Behaviors to Cope with Stress

The five most frequently listed behaviors to cope with stress include (in order) watching television (75%), trying to get the right amount of sleep (73.3%), seeking support from family members (66.7%), listening to music (56.7%), and using humor (50%) (Table 2). Other behaviors mentioned by ≥40% of respondents include exercising regularly, seeking support from co-workers, practicing relaxation, praying, taking naps or rest breaks, doing recreational activities with friends and/or family, and sleeping. 

**Table 2:** Frequency of behaviors to cope with stress.

Behavior	n	Percent
I watch television (TV).	**45**	**75%**
I try to get the right amount of sleep.	**44**	**73.3%**
I seek support from family members.	**40**	**66.7%**
I listen to music.	**34**	**56.7%**
I use humor.	**30**	**50%**
I exercise regularly outside of work (play soccer, walk, jog, bicycling).	29	48.3%
I pray.	29	48.3%
I sleep to cope.	29	48.3%
I take naps or rest breaks.	27	45%
I practice relaxation (spend quiet time alone, take hot showers, *etc.*).	26	43.3%
I do recreational activities with friends and/or family.	25	41.7%
I seek support from people at work.	24	40%
I go shopping.	22	36.7%
I pursue a personal interest activity (gardening, painting).	17	28.3%
I try not to consume drinks that have caffeine or other stimulants.	15	25%
I seek support from friends.	27	25%
I take herbs or vitamins.	13	21.7%
I take medicine prescribed by a doctor.	11	18.3%
I smoke or use other tobacco products to relax.	10	16.7%
I eat lots of bad foods (like chocolate, chips, pizza, pastry, *etc*.).	10	16.7%
I play the lottery.	9	15%
I drink alcohol to relax.	9	15%
I attend dances, social festivals, or other social activities.	7	11.7%
I gamble.	7	11.7%
I get treatment from a massage therapist.	4	6.7%
I get treatment from a chiropractor.	3	5%
I get treatment from a cuarandero or herb doctor.	2	3.3%
I let out my anger on other people.	1	1.7%

When we examined behaviors to cope with stress by demographic and work characteristics, only two bivariate associations were statistically significant (Table 3). A greater percentage of respondents who earned more than $1,000 per month during the past year (94.4%) reported getting the right amount of sleep to cope with stress compared to those earning less than $1,000 (65.0%). A greater percentage of respondents who earned more than $1,000 per month during the past year (88.9%) reported seeking support from family to cope with stress compared to those earning less than $1,000 (60.0%). Income category was chosen by the researchers based on standardized demographic items on instrumentation used with Migrant and Seasonal Farmworkers. There were trends in associations between other demographic and work characteristics and behaviors to cope with stress, although the associations were not statistically significant. The data show that younger respondents reported getting right amount of sleep, listening to music, and watching television more often than older respondents. Also, those who worked ≥8 h per day during the past year reported getting the right amount of sleep, seeking support from family, listening to music, and watching television more often than those working <8 h per day. 

**Table 3:** Top 5 stress coping behaviors and demographic and work characteristics.

Characteristic	Right Amount of Sleep		Support From Family		Listen to Music		Use Humor		Watch Television	
	% (n)	p	% (n)	p	% (n)	p	% (n)	p	% (n)	p
Age (years)		0.223 *		0.593 *		0.229 *		0.881		**0.089** *
20–30	87.5 (14)		75.0 (12)		75.0 (12)		50.0 (8)		93.8 (15)	
≥31–40	71.4 (10)		78.6 (11)		64.3 (9)		50.0 (7)		71.4 (10)	
>40	61.9 (13)		61.9 (13)		47.6 (10)		57.1 (12)		61.9 (13)	
Education (years)		0.818		0.912		0.265		0.788		0.818
<12	73.3 (22)		70.0 (21)		63.3 (19)		46.7 (14)		73.3 (22)	
≥12	76.2 (16)		71.4 (15)		47.6 (10)		42.9 (9)		76.2 (16)	
Total number in household		0.445		0.928		0.626		0.458		0.279
≤5	78.3 (18)		73.9 (17)		56.5 (13)		56.5 (13)		68.3 (18)	
>5	68.2 (15)		72.7 (16)		63.6 (14)		45.5 (10)		63.6 (14)	
Income in past year (monthly)		**0.018**		**0.034** *		0.371		0.695		0.272 *
<$1,000	65.0 (26)		60.0 (24)		62.5 (25)		50.0 (20)		80.0 (32)	
≥$1,000	94.4 (17)		88.9 (16)		50.0 (9)		55.6 (10)		66.7 (12)	
Time in U.S. (years)		0.313		0.975		0.685		0.233		0.991
≤5	80.0 (28)		68.6 (24)		60.0 (21)		57.1 (20)		77.1 (27)	
>5	68.2 (15)		68.2 (15)		54.6 (12)		40.9 (9)		77.3 (17)	
Hours worked (daily) in past year		0.264		**0.088**		0.491		0.940		0.264
<8	64.3 (9)		50.0 (7)		50.0 (7)		50.0 (7)		64.3 (9)	
≥8	79.1 (34)		74.3 (32)		60.5 (26)		51.3 (22)		79.1 (34)	
Part of previous year worked		0.946		0.235		0.515		0.851		0.226 *
All or most	62.5 (15)		79.2 (19)		66.7 (16)		54.2 (13)		83.3 (20)	
≤half	74.2 (23)		64.5 (20)		58.1 (18)		51.6 (16)		67.7 (21)	

* Fisher exact test used due to small cell sizes (chi-square test used for all others).

## 4. Discussion

This study’s findings were similar to those reported by Hovey and Magana, Kim-Godwin and Bechtel and Hiott *et al.*, in which mobile lifestyle, concerns about legal status, financial issues, language barriers, and lack of control over their situation were found to be factors for stress and depression [10,11,12,13,14,15]. The stressors identified in this study related to lack of transportation and inability to communicate in English also confirm the findings of Grzywacz *et al.* [20]. Our results also validate those found by Snipes *et al.* and Hovey, who reported that women in their studies experienced stress and depression regarding their inability to perform traditional household roles [16,17,18,19]. The sub-theme of mental stress related to lack of documentation to work in the U.S. and travel back and forth to and from Mexico or Guatemala to visit family and friends supports the Grzywacz *et al.* research data [20]. It should be noted the researchers included 11 items exploring the construct of depression on the quantitative survey. However—no significance was found in the quantitative portion of the study so was not included in the results. Depression, one of our qualitative findings, is supported by Grzywacz [21] who also found that participants missed their family members back home. Other than this identified issue, depression was not discussed among the participants. This could be due to cultural considerations such as some Latinos do not feel comfortable in openly expressing complex mental health issues [37,38,39,40,45] or due to the group setting of openly sharing very personal and sensitive information.

This study’s research findings were unique in that coping behaviors for stress and depression were examined. Research has focused on stress or depression but rarely how the two correlate, and more importantly, how Latino MSFW cope, either behaviorally or psychologically, with these mental health challenges. Previous research in the general population has shown that stressed or depressed individuals are more likely to pursue negative coping behaviors such as drug or alcohol use, tobacco use, or consumption of a high-fat diet as coping mechanisms, compounding the potential for undesirable consequences, such as domestic violence [16,17,18,22,23,24,25,26,27,28]. Our findings confirmed the use of alcohol and consuming “junk foods” or eating more to deal with stress (15–16% of participants). Conversely, other studies have shown that some individuals deal with stress and depression through positive coping behaviors [29,30]. The current study found that participants also engaged in positive coping behaviors, such as seeking the assistance of a counselor or physician, listening to music, watching TV, shopping, and getting at least 8 hours sleep a night, or taking a hot shower. However, due to the physically rigorous nature of their work, most MSFW are not likely to engage in additional physical activity outside of their job, except for the younger workers who may play an occasional game of soccer. 

### Limitations

One limitation was that some family members attended the two focus groups that were held in community locations. Although this co-attendance at functions by family members is commonplace in the Latino immigrant population, it potentially could have influenced participants’ comments. However some research suggests that this culture may feel more comfortable when family members are present, improving the focus group dynamics [45]. An additional limitation was that more participants were female than a normal sample of Latino farmworkers. This may be due to the focus groups were conducted late in the growing season when many of the male farmworkers had relocated to citrus farming in another state. In addition—this area of North Carolina employs a substantial population of women farmworkers. Focus groups included both genders participating together. If this study were to be implemented again—researchers should consider segregating genders to allow for possible greater disclosure. Another limitation was related to the translation of the focus group discussions. While the translator was fluent in both Spanish and English (which is important to the success of the translation process), and although verbatim transcription was requested, an exact translation is difficult to ensure [45,46]. A third limitation is the self-reported data obtained from the participants, which is subject to their answering questions openly and honestly. To address this limitation, the moderator carefully explained the need for the participants’ candid and honest responses to the questions posed. Specific to the instrumentation—the two items of “seeking support from family or coworkers”—“support” was not defined and left for respondent interpretation. Future studies should define this term for clarity (monetary support, social support, *etc.*). Lastly, for the qualitative analysis, only one member of the research team analyzed the transcriptions to produce the thematic findings. However, the results were discussed with the entire research team for concurrence. Additionally, the researcher is very skilled in qualitative research.

## 5. Conclusions

This study and those mentioned prior suggest that stress and depression are significant mental health issues among Latino MSFW. This population is of particular risk for these health conditions, given their lack of permanent employment, which leads to continued uncertainty and instability. The lack of documentation to work in the U.S. and an inability to travel back and forth to their home countries to visit family and friends was continually identified as a theme related to their experiences of stress and depression. Assistance with documentation issues or temporary residency, such as H2A worker programs, would be valuable for this population to help promote their mental health. In addition, as also confirmed in prior research among Latino MSFWs lack of transportation and licensure to drive were identified as stressors, especially for women. Transportation assistance for this population, beyond just transportation to and from work, should be considered in any programmatic efforts. 

Having a low level of literacy and not speaking English were also sources of stress. Community programs that offer English classes or translational services would help in ameliorating these problems. Mental health services are a valuable resource in rural areas, where those with mental health needs are often underserved. Migrant health centers are key places where mental health counseling programs can be administered. In addition, nontraditional forms of outreach should be considered, such as free Spanish-language mental health service hotlines, worksite counseling, free university programs provided by graduate counseling and psychology students, and evening and weekend hours at community clinics located in or near MSFW communities are needed. 

Negative behaviors for coping with stress and depression, such as alcohol use or over-eating, should be prime targets for intervention. Health education aimed at promoting positive coping behaviors, such as seeking family support, spending time with friends, listening to music, or consulting a trained mental health provider or counselor, should be provided to this population through appropriate community organizations and at cultural events they attend. 

Since research on stress and depression among MSFWs is limited, additional studies need to be conducted to further explore the factors related to these mental health conditions. Exploring differences in stressors based on the locations of the MSFWs’ residences may be of interest. Implementation of evidence-based programs, if available, should be considered. 
